# Variation in gymnemic acid content and non-destructive harvesting of *Gymnema sylvestre* (Gudmar)

**DOI:** 10.4103/0974-8490.72330

**Published:** 2010

**Authors:** Ashok Kumar Pandey, Swati Yadav

**Affiliations:** *Tropical Forest Research Institute (Indian Council of Forestry Research and Education), P.O. RFRC, Mandla Road, Jabalpur - 482 021,M.P., India*

**Keywords:** Cultivation, *Gymnema sylvestre*, gymnemic acid, non-destructive harvesting

## Abstract

**Background:**

Madhunashini (*Gymnema sylvestre* R. Br.) commonly known as ‘Gudmar’ in Hindi is an important medicinal climber and extensively used in almost all Indian System of Medicine as a remedy for diabetes, rheumatism, cough, ulcer, jaundice, dyspepsia, constipation, eyes pain and also in snakebite. In India, it is found growing in Andhra Pradesh, Bihar, Chhattisgarh, Karnataka, Kerala, Madhya Pradesh, Maharastra, Orissa, Tamil Nadu, Uttar Pradesh and West Bengal. The major phytoconstituents are gymnemic acids, gudmarin and saponins.

**Methods:**

In the present study, *Gymnema* germplasm collected from various regions of Madhya Pradesh was evaluated on the basis of their morphological characteristics and gymnemic acid content. Gymnemic acid content in the leaves was estimated by HPLC. We have also standardized the non-destructive harvesting practices of Gudmar. Selective harvesting was done without harming the main plant. Only mature leaves (60%) were hand plucked in the month of October. Second harvest was done in the month of June.

**Results:**

Data revealed that gymnemic acid content varied between 0.96% ± 0.03 (Seoni) to 1.58% ±0.03 (Amarkantak). It was also observed that the leaves left at the time of 1^st^ harvest during October matured in June at the time of 2^nd^ harvest.

**Conclusion:**

Non destructive harvesting practice did not have any negative impact on overall development of the plant. It is evident that there is wide variation in the morphological characteristics and gymnemic acid content in *G. sylvestre* collected from various locations, which can be exploited for further crop improvement programmes.

## INTRODUCTION

More than 80% of the world’s population uses natural medicines and depends on medicinal plants for health care. In recent years, the growing demand for herbal products has led to a quantum jump in volume of plant materials traded within and across the countries. At present, 90% collection of herbal raw drugs used in the manufacture of Ayurveda, Siddha, Unani, and Homeopathy systems of medicine is largely from the wild out of which 70% collection involves destructive harvesting. Due to this spurt, medicinal plants are being overexploited and many of them are pushed to the brink of extinction. Many medicinal plants are highly sensitive to the level of harvest and fragility of the ecosystem; one of them is Gudmar.

**Figure 1:**
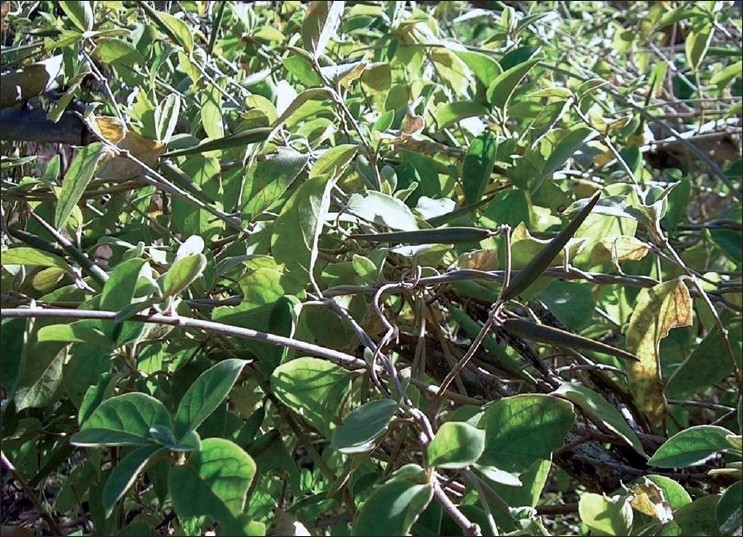
*Gymnema sylvestre* (Gudmar) plant

Madhunashini (*Gymnema sylvestre* R. Br.) commonly known as “Gudmar” in Hindi is an important medicinal climber belonging to the family Asclepiadaceae. In India, it is found in the forests of Andhra Pradesh, Bihar, Chhattisgarh, Karnataka, Kerala, Madhya Pradesh, Maharastra, Orissa, Tamil Nadu, Uttar Pradesh, and West Bengal. Due to its heavy demand in South East Asian countries, the plant is becoming endangered and under cultivation in southern states of India, particularly in Tamil Nadu.[1] The leaves are simple, opposite, elliptic, or ovate and hairy; the flowers are small, yellow, and in umbellate cymes. The follicles are terete, lanceolate, and upto 7.5 cm in length [Figure 1]. Leaves and roots are the useful parts of the plant. This climber is extensively used in almost all the Indian system of medicine as a remedy for rheumatism, cough, ulcer, and pain in eyes. It is also useful in inflammations, dyspepsia, constipation, jaundice, etc. Roots have been reported as a remedy for snakebite.[2,3] The plant is popularly known as “Gudmar” for its distinctive property of temporarily destroying the taste of sweetness. The leaves of this plant have been used for over 2000 years to treat diabetes, giving it a prominent place in the indigenous system of medicines in this country. Administration of Gudmar leaves lowers the blood glucose level in diabetic patients[4] and the alcoholic extracts of *Gymnema* leaves have been shown to increase the release of insulin from pancreatic β-cells.[5] Studies have revealed that the water extract of the leaves of the plant inhibited absorption of glucose in the small intestine and suppressed the increase in blood sugar level after administration of sucrose in rats. The ethanolic extract of the leaves demonstrated antimicrobial activity against *Bacillus pumilis, B. subtilis, Pseudomonas aeruginosa*, and *Staphylococcus aureus* and inactivity against *Proteus vulgaris* and *Escherichia coli*.[6] Antiallergic, antiviral, lipidȔlowering, and other effects of the plant parts are also reported by Porchezhian and Dobrial.[7] The major phytoconstituents are gymnemic acids (GA), Gudmarin, a polypeptide of 35 amino acids, and saponins.[3] Leaves contain several *O*-iso-propylidene derivatives of gymnemagenin, a hexahydro-terpene, crystalline gymnemagenin, gymnestrogenin 1,2, and GA (the antisweet principle), which is a complex mixture of atleast nine closely related acidic glycosides.[7] Application of GA on the tongue depresses the taste of sucrose in man.[8] The plant leaves are used to treat type II (adult-onset) diabetes.[9]

The quantity of GA, the active principle in *Gymnema* leaves is variable among accessions from different ecoclimatic regions.[10] Considerable variations exist among the morphological traits of *Gymnema* accessions from Tamil Nadu and Kerala.[11,12] However, detailed information on the extent of variability in the *Gymnema* populations of Madhya Pradesh is not available. Hence, a study was undertaken to characterize the morphological and GA variations among the *Gymnema* germplasm accessions from diverse ecoclimatic regions of Madhya Pradesh.

Villagers collect the leaves of Gudmar from the forest area and sell in the market. Normally collectors harvest the leaves by using destructive harvesting methods by cutting the main stem and pulling down to the ground. Due to this prevailing practice, the availability of Gudmar is decreasing day by day. Till now all the collection is coming from the wild and the species is becoming vulnerable. Keeping the above into consideration, we conducted a study to develop a non-destructive harvesting practice for Gudmar.

## MATERIALS AND METHODS

### Evaluation of germplasm

*Gymnema* germplasm was collected from four locations of Madhya Pradesh, India, during 2003 and maintained at Non Wood Forest Produce (NWFP) Nursery of the Tropical Forest Research Institute, Jabalpur. The plants were grown under open conditions at 0.5 × 0.5 m spacing and the study was conducted for 3 years after planting. The vegetative traits such as habit, leaf length, leaf width, and leaf shape were used for morphological characterization. Regular observations were taken to find out the morphological variations among different accessions. GA concentration in the leaves was estimated by HPLC.[13] Leaf samples of Gudmar for GA estimation were collected during February and March 2005. Leaves were collected from each plant such that the sample contained both tender and mature leaves.

### HPLC determination

Chromatographic equipment and conditions

A Waters (Milford, USA) gradient HPLC instrument equipped with two 515 pumps and controlled by an interface module PC2, manual injector valve (Rheodyne), reverse phase C18 (100 × 4.6 mm i.d.) X bridge HPLC column (Waters, Milford, USA) and Waters 2996 PDA (Photo Diode Array) detector was used for HPLC analysis. Waters Empower software was used to control the equipments and for the analysis of data. The solvents were prefiltered by a Millipore filtration unit (Millipore, Billerica, MA).

The other operating conditions were as follows:

**Table:** 

Eluent	Acetonitrile — water (80:20)
Flow — rate	1.0 ml/minute
Detector	PDA
Wavelength	210 nm
Amount injection	20 µl

### Chemicals and standard solutions

Standard gymnemagenin (98% pure) was obtained from M/s Natural Remedies, Bangalore, India. All chemicals and reagents used were of HPLC grade. The reference solution of gymnemagenin was prepared by dissolving 10 mg of standard in 5 mL of 50% (v/v) aqueous methanol.

### Sample preparation

**Figure 2:**
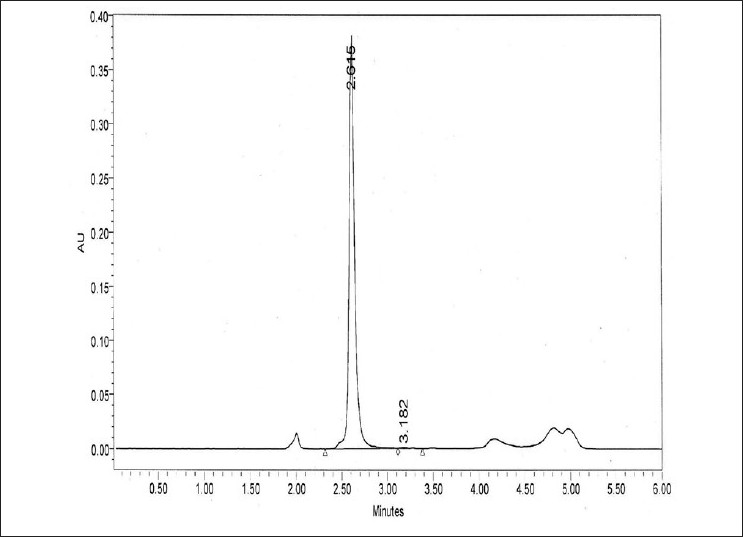
HPLC chromatogram of standard gymnemic acid

Leaves of *G. sylvestre* were collected from the nursery for estimation of GA. The leaf samples were prepared and analyzed according to the method of Toshihiro *et al*. 1994.[13] One gram of powdered leaves of *G. sylvestre* was weighed accurately into a round-bottom flask, 50 mL of 95% (v/v) aqueous methanol was added, the mixture was refluxed on a water bath for 30 min, and then filtered. Another 50 mL of 95% methanol was added; the sample was refluxed for a further 30 min and filtered. The alcoholic extracts were combined and evaporated on a rotary evaporator. To the resulting semi-solid, 5 mL of 50% (v/v) methanol and 1 mL of potassium hydroxide solution (containing 11 g/100 mL water) were added and the mixture was refluxed on a water bath for 1 h. The mixture was cooled and the whole solution was again refluxed for 1 h after adding 0.9 mL of concentrated hydrochloric acid. The resulting solution was cooled, adjusted to pH 7.5—8.5 with potassium hydroxide solution, made up to a volume of 50 mL with 50% (v/v) methanol and filtered through Whatman no. 1 filter paper. The filtrate was subjected to HPLC analysis. The amount of gymnemagenin was determined from the calibration curve obtained by plotting the concentration of standard against the peak area on the scanned chromatogram. HPLC chromatogram of standard gymnemic acid is given in Figure 2.

Experiments were laid out to standardize non-destructive harvesting method of Gudmar in NWFP Nursery of the institute during 2003—05. In the experiments, selective harvesting methods were used. All the leaves were not harvested at a time. Only mature leaves (60%) were hand plucked in the month of October and young immature leaves were left on the plant. Second harvest was done in the month of June. It was observed that the leaves left at the time of first harvest during October matured in June at the time of second harvest. This method did not affect the growth of the plant.

## RESULTS AND DISCUSSION

### Gymnemic acid determination

**Figure 3:**
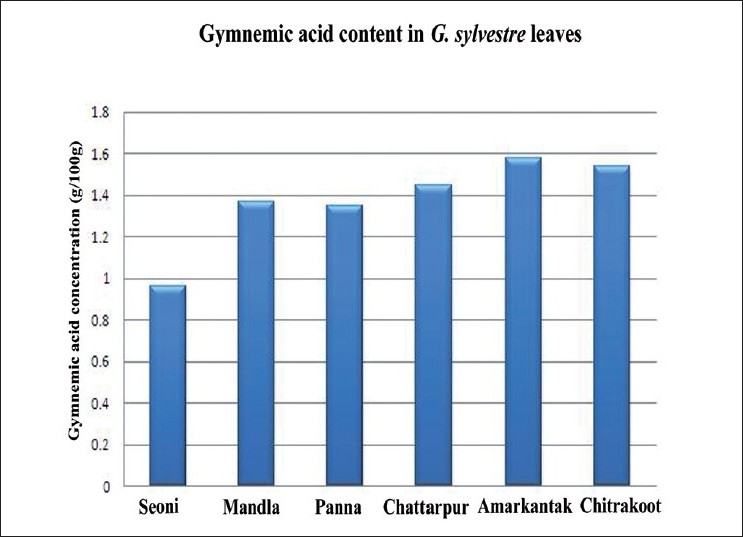
Variation in gymnemic acid content in *Gymnema selvestre*

HPLC quantification revealed that GA concentration in the leaves collected from various places varied between 0.96% (Seoni) and 1.58% (Amarkantak) [Table 1] on dry weight basis. The accession collected from Chitrakoot contains 1.54% GA, which is at par with the Amarkantak accession. The accessions from Mandla (1.37%), Panna (1.35%), and Chhatarpur (1.45%) have almost similar GA content. The extent of variability in the *Gymnema* populations goes from Seoni to Amarkantak in Madhya Pradesh. Graphical representation of GA variation is given in Figure 3. The GA being secondary metabolites are often influenced by the environmental and seasonal factors. From the results, it is evident that there is wide variation in the morphological characters and GA content of Gudmar collected from different locations, which can be further exploited to popularize the useful genotypes for the extraction of drugs. Our findings are corroborated with the findings of Yokota *et al*., Thamburaj *et al*., and Nair and Keshavachandran.[10–12]

**Table 1:** Variation in leaf size and gymnemic acid in *Gymnema selvestre* collected from various places

Treatment	Leaf length (cm)	Leaf width (cm)	Gymnemic acid (g/100g)
Seoni	4.45	2.65	0.96
Mandla	4.71	2.66	1.37
Panna	3.58	1.67	1.35
Chhatarpur	4.61	2.64	1.45
Amarkantak	4.88	2.68	1.58
Chitrakoot	4.92	2.65	1.54
SE±	0.07	0.06	0.03
CD at 5%	NS	NS	NS

Results showed that all the accessions studied exhibit the habit of woody climbers. Warrier *et al*. also described Gudmar as a large, much branched woody climber.[14] All other characters, however, showed great variations. For instance, leaf shapes included elliptic-oblong, ovate, ovate-lanceolate, lanceolate, and cordate. Average leaf length ranged from 3.58 (Panna) to 4.92 cm (Chitrakoot) and the average leaf width varied from 1.67 (Panna) to 2.68 cm (Amarkantak). Most of the leaves studied were hairy. However, some leaves collected from Panna were nonhairy. The leaf base shape was mostly subcordate, but truncate, rounded, and obtuse were also seen. The leaf tip shape in Gudmar was either acute or acuminate. However, a few accessions showed cuspidate shape. Similar observations were made earlier by Thamburaj *et al*. in a study involving 12 germplasm accessions.[12] They observed lanceolate and ovate shapes with the leaf tip being either blunt or pointed and 50% of their genotypes were highly pubescent while the others were nonhairy.

### Non-destructive harvest

The data on non-destructive harvesting method of Gudmar was recorded and tabulated in Table 2. During first harvest, in the month of October, 751.66 leaves were harvested and 231.00 young leaves were left for maturity. In second harvesting, 667.33 leaves were harvested and 192.00 leaves were left. In this way, a total of 1446.00 leaves were harvested in a year. On an average 200—500 g dried leaves per plant can be obtained from a 4-year-old plant yielding about 4,000—6,000 kg of dried leaves per hectare. The crop can be cultivated for 10—15 years under good management.

**Table 2:** Non-destructive harvesting of *Gymnema selvestre* (Gudmar) leaves

First harvesting			Second harvesting			
Total no of leaves	No. of leaves harvested	No. of leaves left	Total no of leaves	No. of leaves harvested	No. of leaves left	Total no. of leaves harvested in year
961.66	751.66	231.00	859.33	667.33	192.00	1446.00

### CONCLUSION

It can be concluded that the variation exists among the different accessions of *G. sylvestre*, which can be utilized for the production of quality herbal formulations. The same can also be utilized for further crop improvement programs to obtain quality raw material. The non-destructive harvesting method will be an efficient tool for sustainable management of *G. sylvestre*. The leaves should be selectively harvested after 2 years of plantation by hand plucking twice in the year (June and October).

## References

[1] FRLHT (1997). Medicinal plants of India. Guidelines for National Policy and Conservation Programmes.

[2] Anonymous (1995). A Compendium of 500 species. Indian Medicinal Plants.

[3] Nadkarni KM (1993). Indian Materia Medica.

[4] Shanmugasundaram KR, Panneerselvam C, Samudram P, Shanmugasundaram ER (1981). The insulinotropic activity of *Gymnema sylvestre* R.Br. An Indian medicinal herb used in controlling diabetes mellitus.

[5] Shanmugasundaram ER, Gopinath KL, Shanmugasundaram KR, Rajendran VM (1990). Possible regeneration of the islets of langerhans in streptozotcin- diabetes rats given *Gymnema sylvestre* leaf extracts. J Ethnopharmcol.

[6] Satdive RK, Abhilash P, Fulzele DP (2003). Antimicrobial activity of *Gymnema sylvestre* leaf extract. Fitoterapia.

[7] Porchezhian E, Dobriyal RM (2003). An overview on the advances of *Gymnema sylvestre*: Chemistry, pharmacology and patents. Pharmazie.

[8] Glaser D, Hellekant G, Brouwer JN, Wel H Van der (1984). Effects of gymnemic acid on sweet taste perception. Chem Senses.

[9] Singh VK, Umar S, Ansari SA, Iqbal M (2008). *Gymnema sylvestre* for Diabetics. J Herbs Spices Med Plants.

[10] Yokota T, Mizutani K, Okada K, Tanak O (1994). Quantitative analysis of gymnemic acids by high performance liquid chromatography. J Jpn Soc Food Sci Technol.

[11] Thamburaj S, Subbaraj D, Kasturi S, Vijayakumar M (1996). Evaluation of germplasm accessions of *Gymnema sylvestre* R.Br. Indian Horticulture.

[12] Nair S, Keshavachandran R (2006). Genetic variability of chakkarakolli (*Gymnema sylvestre* R.Br) in Kerala assessed using morphological and biochemical markers. J of Tro Agri.

[13] Toshihiro Y, Kenzi M, Kenzo O, Osamu T (1994). Quantitative analysis of gymnemic acids by high performance liquid chromatography. Nipp Shok Kag Kai.

[14] Warrier PK, Nambiar VP, Ramankutty C (1995). Indian Medicinal Plants. A compendium of 500 species.

